# High Expression of GABA_A_ Receptor β Subunit Genes Is Associated with Longer Overall Survival in Medulloblastoma

**DOI:** 10.3390/brainsci14111146

**Published:** 2024-11-15

**Authors:** Jander M. Monteiro, Matheus Dalmolin, Marcelo A. C. Fernandes, Jaqueline I. R. Ramos, Carmen A. P. M. Ribas, Fernando I. Tabushi, Rafael Roesler, Gustavo R. Isolan

**Affiliations:** 1 Graduate Program in Principles of Surgery, Mackenzie Evangelical University, Curitiba 80730-000, Brazil; jandermmonteiro@gmail.com (J.M.M.);; 2 InovAI Lab, nPITI/IMD, Federal University of Rio Grande do Norte, Natal 59078-970, Brazil; 3 Bioinformatics Multidisciplinary Environment (BioME), Federal University of Rio Grande do Norte, Natal 59078-970, Brazil; 4 National Science and Technology Institute for Children’s Cancer Biology and Pediatric Oncology—INCT BioOncoPed, Porto Alegre 90035-003, Brazil; 5 Department of Computer Engineering and Automation, Federal University of Rio Grande do Norte, Natal 59078-970, Brazil; 6 Ribeirão Preto School of Dentistry, University of São Paulo, Ribeirão Preto 14040-904, Brazil; 7 Department of Pharmacology, Institute for Basic Health Sciences, Federal University of Rio Grande do Sul, Porto Alegre 90035-003, Brazil; 8 Cancer and Neurobiology Laboratory, Experimental Research Center, Clinical Hospital (CPE-HCPA), Federal University of Rio Grande do Sul, Porto Alegre 90035-003, Brazil; 9 The Center for Advanced Neurology and Neurosurgery (CEANNE), Porto Alegre 90560-010, Brazil

**Keywords:** γ-aminobutyric acid, GABA_A_ receptor, GABA_A_ β subunit, medulloblastoma, pediatric brain cancer

## Abstract

**Background/Objectives:** Most of the rapid inhibitory neurotransmission in the brain is mediated through activation of the γ-aminobutyric acid (GABA) type A (GABA_A_) receptor, which is a ligand-gated ion channel. GABA_A_ receptor activation via GABA binding allows for an intracellular influx of Cl^−^ ions, thus inducing cellular hyperpolarization. Each GABA_A_ receptor consists of a combination of five subunits, and several subunits have been proposed as biomarkers and therapeutic targets in cancer. Here, we show the expression of genes encoding β subunits of the GABA_A_ receptor, namely *GABRB1*, *GABRB2*, and *GABRB3*, across the four different molecular subgroups of medulloblastoma (MB), which is the most common malignant pediatric brain tumor. We also show the associations of GABA_A_ receptor β subunits with MB patients’ overall survival (OS). **Methods:** The expression of genes encoding GABA_A_ receptor β subunits was analyzed using a previously described dataset comprising 763 MB tumor samples. Patients were classified into high- and low-gene-expression groups, and the Kaplan–Meier estimate was used to examine the relationship between gene expression levels and patient OS. **Results:** High *GABRB1* expression was associated with better OS within each of the four molecular subgroups. The *GABRB2* gene showed higher transcript levels in Group 3 MB compared to all other subgroups, and high expression was associated with better prognosis in Group 3 tumors. *GABRB3* expression was significantly higher in Group 3 and Group 4 MB, and high expression of *GABRB3* genes was associated with longer OS in the sonic hedgehog (SHH) subgroup. The high expression of *GABRB1*, *GABRB2*, and *GABRB3* is associated with longer patient OS in a subgroup-specific manner. **Conclusions:** These results indicate a role for GABA_A_ receptors containing β subunits in influencing MB progression.

## 1. Introduction

The γ-aminobutyric acid (GABA) type A (GABA_A_) receptor mediates most of the rapid inhibitory neurotransmission in the central nervous system (CNS). Each GABA_A_ receptor consists of a combination of five subunits drawn from a repertoire of 19 proteins (α1-6, β1-3, γ1-3, δ, ε, θ, π, ρ1-3). Most of the functional GABA_A_ receptors consist of two α, two β and one γ or δ subunit, which, when combined, form a ligand-gated chloride (Cl^−^) ion channel activated by GABA binding [1,2]. GABAergic transmission regulates cerebellar development, which is critical for the establishment of connections between basket cells and Purkinje cells [3], and GABA influences the differentiation and proliferation of cerebellar NG2-expressing oligodendrocyte precursor cells [4]. Mouse models with reduced GABAergic function show impaired developmental synapse selection in the cerebellum [5]. Developmental changes in the subunit composition of GABA_A_ receptors may be involved in the GABA modulation of cerebellar formation [6].

Several genes involved in normal cerebellar development may also play a role in oncogenesis [7,8,9,10,11,12]. Medulloblastoma (MB), the most common malignant brain tumor affecting children, likely arises from neuronal progenitors in the cerebellum and some subtypes of MB tumors appear arrested at different developmental stages [7,8]. MB is currently classified into four consensus molecular subgroups, namely wingless-activated (WNT), sonic hedgehog (SHH), Group 3, and Group 4, with Group 3 MB being particularly associated with poorer survival and metastasis [13,14,15].

The gene expression patterns in different molecular subgroups of MB resemble those found in cerebellar cell types that are temporally restricted over the course of development [12]. Transitional cerebellar progenitors that connect neural stem cells to neuronal lineages in the developing cerebellum are enriched in more aggressive MB subgroups [10]. Increased expression of the *GABRA5* gene, which encodes the α5 subunit of the GABA_A_ receptor, was reported in Group 3 MB, and pharmacological activation with the specific α5-GABA_A_ receptor agonist QHii066 reduces the survival of MB cells, suggesting that increased GABA_A_ receptor activity can counteract MB growth [16]. However, the role of β subunits remains poorly understood. Here, we analyzed the transcript levels of genes encoding β subunits of the GABA_A_ receptor, namely *GABRB1*, *GABRB2*, and *GABRB3*, across the four different molecular subgroups of MB, and their association with patient prognosis as assessed by OS.

## 2. Materials and Methods

### 2.1. Gene Expression, Tumor and Patient Data

The expression of genes encoding GABA_A_ receptor β subunits was analyzed using the transcriptome dataset, comprising 763 tumor samples from patients with MB, who constituted the Cavalli cohort [17]. We selected 612 samples that included complete survival data, specifically, OS time and survival status (alive or dead). Samples lacking complete survival information were excluded, as these data are crucial for Kaplan–Meier analysis. The selected 612 tumor samples were distributed according to molecular subgroup: Group 3, *n* = 113; Group 4, *n* = 264; SHH, *n* = 172; WNT, *n* = 64. Expression data were processed and normalized by the authors of the original study and are publicly available in the GEO database under the accession code GSE85218.

### 2.2. Statistical Analysis

Wilcoxon tests were used to analyze differences in the expression of genes encoding the β subunit of the GABA_A_ receptor (*GABRB1*, *GABRB2*, *GABRB3*) among MB subgroups (WNT, SHH, Group 3, and Group 4) as the data did not follow a normal distribution. Normality was assessed using the Shapiro–Wilk test, which is particularly suited for smaller sample sizes. This test evaluates the null hypothesis that a sample comes from a normally distributed population. A *p*-value < 0.05 in the Shapiro–Wilk test indicated a significant deviation from normality in our dataset. Pairwise comparisons were conducted using the Dunn test with Holm-adjusted *p*-values for multiple testing corrections. These analyses were performed using the ‘ggstatsplot’ package.

To assess the association between gene expression levels for *GABRB1*, *GABRB2*, and *GABRB3* and patient OS, we used the Kaplan–Meier estimate. Patients were classified into high- and low-gene-expression groups using the ‘Survminer’ package. The surv_cutpoint() function from Survminer was employed to determine the optimal cutoff point for gene expression that maximizes the difference in survival between the groups. The parameter minprop = 0.2 was set to ensure that at least 20% of the patients were included in the smaller group, preventing a highly unbalanced division. This process objectively identifies the most appropriate threshold for high and low expression based on the data. Survival analysis was then performed using the ‘Survival’ package, with *p* < 0.05 indicating significant differences between groups.

## 3. Results

### 3.1. Expression of GABRB1, the Gene Encoding the β1 Subunit of the GABAA Receptor, in Molecular Subgroups of MB Tumors

**Figure 1:**
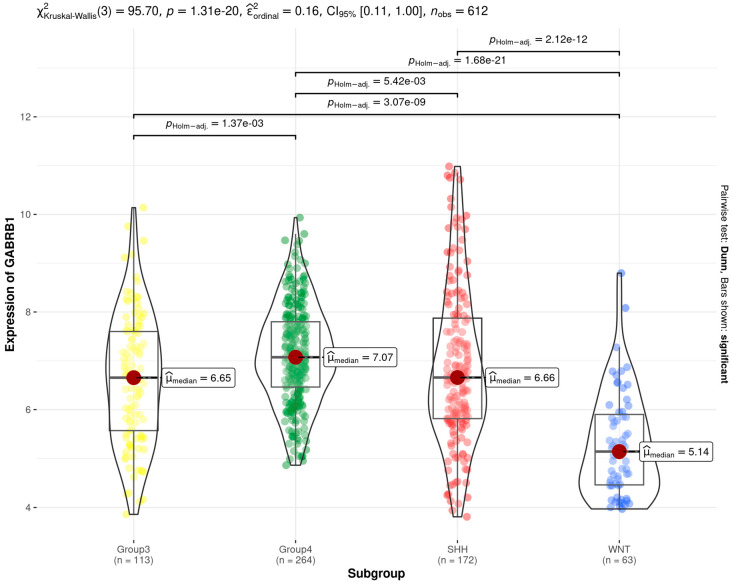
Gene expression of *GABRB1*, the gene encoding the β1 subunit of the GABA_A_ receptor, in different molecular subgroups of human MB. Tumors from the dataset described by Cavalli et al. [17]. Results are presented in boxplot format as log2-transformed signal intensity. Bars show data for Group 3 (*n* = 113), Group 4 (*n* = 264), SHH (*n* = 172), and WNT (*n* = 63) MB; *p* values are indicated in this figure.

The expression of the *GABRB1* gene in Group 4 MB was significantly higher compared to that in Group 3, SHH, and WNT MB. Conversely, expression in the WNT subgroup was lower compared to all other molecular subgroups (Figure 1).

### 3.2. Association Between GABRB1 Expression and Patient Survival in Different Molecular Subgroups of MB

**Figure 2:**
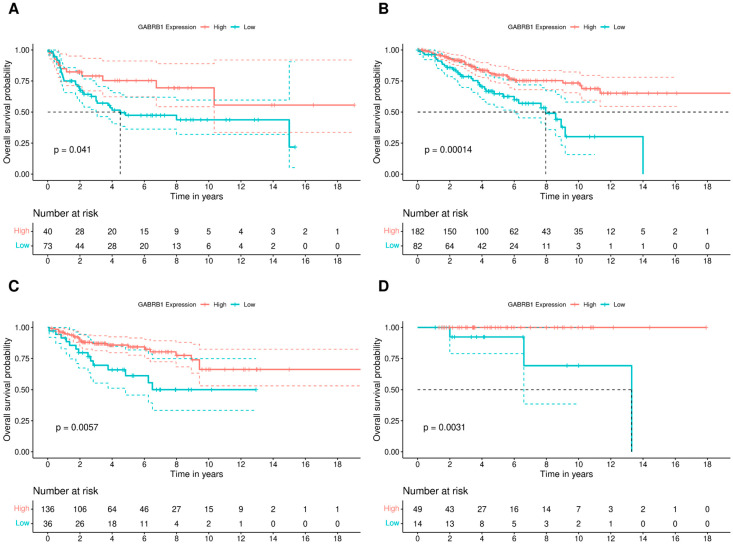
Gene expression of the *GABRB1* β GABA_A_ receptor subunit is associated with better prognosis in patients with MB. (**A**) OS analysis of patients with Group 3 MB tumors (*n* = 113), comparing high (*n* = 40) and low (*n* = 73) *GABRB1* expression. (**B**) OS analysis of patients with Group 4 MB tumors (*n* = 264), comparing high (*n* = 182) and low (*n* = 82) expression of *GABRB1*. (**C**) OS analysis of patients with SHH MB tumors (*n* = 172), comparing high (*n* = 136) and low (*n* = 36) *GABRB1* expression. (**D**) OS analysis of patients with WNT MB tumors (*n* = 63), comparing high (*n* = 49) and low (*n* = 14) *GABRB1* expression. In all plots, red lines represent patients with high *GABRB1* expression, and blue lines represent patients with low expression. The number of patients at risk at each time point is shown below each plot. Data show analyses of MB tumors in the dataset described by Cavalli et al. [17]; *p* values are indicated in this figure.

For each MB subgroup, we then conducted survival analyses to verify whether *GABRB1* expression was associated with patient prognosis. A higher expression of *GABRB1* was significantly associated with better prognosis, as indicated by longer OS, in all MB subgroups (Figure 2).

### 3.3. Expression of GABRB2 and GABRA3 and Subgroup-Specific Association with Patient Survival in MB

**Figure 3:**
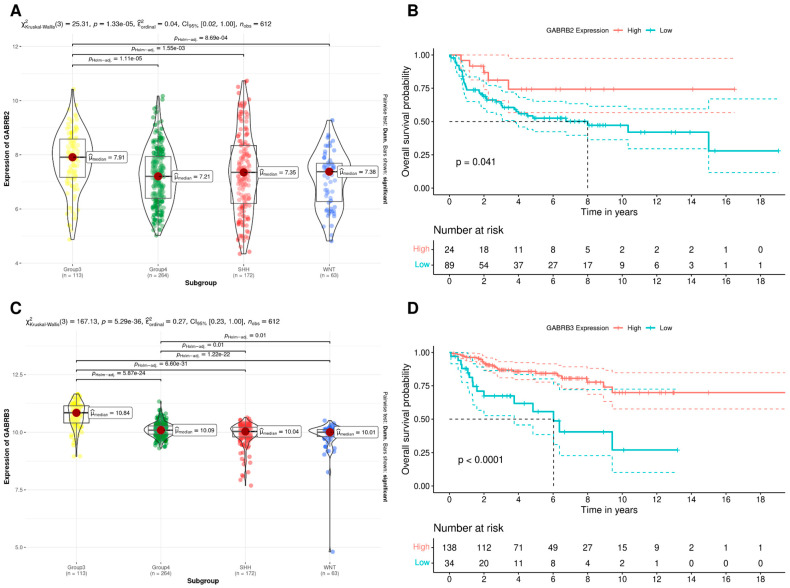
Gene expression of the *GABRB2* and *GABRB3* β GABA_A_ receptor subunits is associated with better prognosis in specific molecular subgroups of MB. (**A**) *GABRB2* expression in molecular subgroups of MB, including Group 3 (*n* = 113), Group 4 (*n* = 264), SHH (*n* = 172), and WNT (*n* = 63) tumors, shown as violin plots. (**B**) OS analysis of patients with Group 3 MB tumors, comparing high (*n* = 24) and low (*n* = 89) *GABRB2* expression. (**C**) *GABRB3* expression in molecular subgroups of MB, shown as violin plots. (**D**) OS analysis of patients with SHH MB tumors, comparing high (*n* = 138) and low (*n* = 34) *GABRB3* expression. In the Kaplan–Meier plots (**B**,**D**), red lines represent patients with high *GABRB* expression, and blue lines represent those with low expression. The number of patients at risk at each time point is shown below each plot; *p* values are indicated in this figure.

The *GABRB2* gene showed significantly higher expression in Group 3 MB compared to all other subgroups (Figure 3A), and high *GABRB2* expression was associated with better prognosis within Group 3 tumors (Figure 3B). *GABRB3* expression was significantly higher in Group 3 and Group 4 MB compared to the SHH and WNT subgroups (Figure 3C). High expression of the *GABRB3* gene was associated with better prognosis in SHH MB (Figure 3D).

## 4. Discussion

Our findings show that the genes encoding β subunits of the GABA_A_ receptor are expressed across all four molecular subgroups of MB. Higher transcript levels were associated with better patient survival in all MB subgroups for *GABRB1* and in a subgroup-specific manner for *GABRB2* and *GABRB3*. The expression of *GABRB2* was higher and associated with better prognosis in Group 3 tumors, whereas *GABRB3* showed higher levels in Group 3 and Group 4 MB, but was related to longer survival in the SHH subgroup.

We previously reported that high levels of *GABRA2*, *GABRA3*, *GABRB3*, *GABRG1*, and *GABRG2* indicated better prognosis in glioma, and *GABRB3* was particularly associated with longer OS in patients with lower-grade gliomas [18]. Together with these previous findings, our present results support the possibility that GABAergic neurotransmission mediated by GABA_A_ receptors can impair the growth of brain tumors. Consistent with this view, the pharmacological stimulation of GABA_A_ receptors has been shown to display inhibitory effects in experimental MB through mechanisms that may involve *TP53* upregulation [16,19]. These findings have potential clinical implications, suggesting the possibility that drugs that stimulate GABAA receptors, such as benzodiazepines, could be used as adjuvant treatment options in MB patients.

The mechanisms underlying GABAergic involvement in brain tumors remain unclear. GABA_A_ receptors are critical for synapse selection during development in the cerebellum, indicating that alterations in the expression or function of GABA_A_ receptors could lead to abnormal cerebellar formation related to MB carcinogenesis [4,5,6]. The change in cell membrane potential that follows GABA_A_ receptor activation leads to a variety of intracellular signaling stimuli partially related to Ca^2+^ mobilization. GABA_A_ receptor activation can result in neuronal inhibition, or in the development of neurons with altered Cl^−^ levels, excitation [20,21,22]. GABA induces the phosphorylation and activation of histone H2AX through the phosphoinositide 3-kinase (PI3K) pathway to reduce stem cell proliferation [23,24]. GABA_A_ stimulation by a benzodiazepine leads to the upregulation of tumor-suppressing genes *MDM2*, *PTEN*, and *TP53*, and also of *AKT1-3*, in MB cells [19].

A previous analysis of the Cavalli cohort by Kallay et al. showed high *GABRB3* expression across all four MB molecular subgroups and indicated that the gene expression pattern of different subunits of the GABA_A_ receptor was consistent with the assembly of functional receptors. The different molecular subgroups of MB display distinct GABA_A_ receptor subunit expression signatures, which might imply subgroup-specific regulatory actions of GABAergic transmission on MB tumor progression [19].

Evidence from studies investigating peripheral solid tumors also supports the involvement of GABA_A_ β subunits and their encoding genes as biomarkers or therapeutic targets in cancer [25,26,27]. For example, the expression of *GABRB2* and *GABRB3* is associated with colon adenocarcinoma occurrence, and a low expression of *GABRB1* correlates with patient survival in that cancer type [28]. GABA_A_ β3 subunit content is higher in triple-negative breast cancer (TNBC) in comparison to non-tumor cells, and pharmacological antagonism or genetic knockdown of that subunit reduces cell proliferation and migration, induces cell cycle arrest, and increases p21 expression in TBNC cells [29]. The GABA_A_ β3 subunit is highly expressed in hepatocellular carcinoma (HCC) cancer stem cells (CSCs) compared to normal stem cells, and this may partially account for HCC CSC’s depolarized state and responses to GABA_A_ receptor agonists [30].

## 5. Conclusions

This study used an established MB dataset to provide early gene expression evidence suggesting a role for β subunit-containing GABA_A_ receptors in tumor aggressiveness. Our results show that higher transcript levels of *GABRB1*, *GABRB2*, and *GABRB3* are associated with longer patient OS. The modulatory role of GABA_A_ receptors in brain tumors highlights the importance of exploring the influence of neuronal cell surface receptors that function as neurotransmitter-gated ion channels on brain cancer progression.

## Data Availability

The dataset analyzed in this study is available in the Gene Expression Omnibus repository, https://www.ncbi.nlm.nih.gov/geo/query/acc.cgi?acc=GSE85217.
